# Performance and reliability of large language models on the European Board of Hand Surgery examination: a multi-model evaluation study

**DOI:** 10.1177/17531934261436305

**Published:** 2026-04-21

**Authors:** Ibrahim Güler, Lindsay Muir, Gerrit Grieb, Philipp Moog, Armin Kraus, Henrik Stelling

**Affiliations:** 1 Department of Health Management, Friedrich Alexander University Erlangen-Nuremberg FAU, Germany; 2 Department of Plastic Surgery and Hand Surgery, Gemeinschaftskrankenhaus Havelhöhe, Germany; 3 Department of Plastic, Aesthetic and Hand Surgery, University Hospital Magdeburg, Germany; 4 Department of Hand and Plastic Surgery, Salford Royal Hospital, UK; 5 Examination Committee, European Board of Hand Surgery, Federation of European Societies for Surgery of the Hand; 6 Department of Plastic Surgery and Hand Surgery, Medical Faculty, RWTH Aachen University, Germany; 7 Department of Plastic Surgery and Hand Surgery, Klinikum Rechts der Isar, Technische Universität München, Germany; 8 Department of Nuclear Medicine, Klinikum Ernst von Bergmann, Germany; 9 Practices for Nuclear Medicine, Germany

**Keywords:** Artificial intelligence, diploma examination, large language models, medical education, hand surgery, board certification, European Board of Hand Surgery

## Abstract

**Introduction:**

Artificial intelligence (AI) has demonstrated transformative potential in medical education and assessment, with large language models achieving competitive results across multiple high-stakes examinations. In this study, we evaluated the performance and inter-run reliability of 10 widely adopted large language models (LLMs) on the European Board of Hand Surgery written examination.

**Methods:**

Ten LLMs were assessed on the complete 300-item European Board of Hand Surgery written examination using standardized zero-shot prompting. The models included five proprietary systems (GPT-5 Pro, Claude Sonnet 4.5, Gemini 2.5 Pro, Grok-4 and ERNIE 4.5 Turbo) and five open-source architectures (DeepSeek V3.2, Qwen3 Max, Mistral Medium 3.1, Llama 3.3 and Falcon H1). Each LLM completed five independent runs, producing 15000 answers analysed for mean accuracy, 95% confidence intervals and inter-run reliability using Cohen’s kappa (*κ*).

**Results:**

Mean accuracy across the LLMs ranged from 72 to 85%, corresponding to total European Board of Hand Surgery scores between 131 and 211 points. Seven of the 10 LLMs reached or exceeded the illustrative pass threshold of 75%, equivalent to 150 of 300 points. Proprietary systems showed consistently higher mean accuracy than open-source systems. The highest-performing LLM (GPT-5 Pro) achieved 85% accuracy with a 95% confidence interval of 84 to 86% and a mean inter-run reliability measured by Cohen’s *κ* of 0.739. The overall reliability across the LLMs was 0.821.

**Conclusions:**

Contemporary LLMs show robust and reproducible performance on a complex surgical certification examination, with proprietary architectures tending to outperform open-source counterparts. Although several models reached or exceeded an illustrative pass threshold, persistent gaps in subspecialty knowledge remain such as congenital anomalies and complex reconstructions. Therefore, LLMs may assist in structured learning and examination preparation but require specialist oversight and remain unsuitable for independent subspecialty decision-making.

**Level of evidence:**

Not applicable.

## Introduction

Artificial intelligence (AI) has demonstrated transformative potential in medical education and assessment, with large language models (LLMs) achieving competitive results across multiple high-stakes examinations. Recent research has shown that these models can approach or even exceed human-level accuracy on written medical licensing tests such as the US Medical Licensing Examination, national board examinations, and specialty-specific assessments (Gilson et al., 2023; Gordon et al., 2024; Kung et al., 2023; Lee et al., 2021; Liu et al., 2024; Ouanes and Farhah, 2024; Takagi et al., 2023). These advances have intensified academic interest in the role of LLMs for knowledge evaluation, clinical reasoning assessment, and medical education.

The European Board of Hand Surgery (EBHS), administered by the Federation of European Societies for Surgery of the Hand, is recognized across Europe and described in international guidance and candidate reports (Calcagni, 2018; Nachemson, 2000). To date, evidence indicates modest accuracy and considerable variability among LLMs on EBHS content. In recent evaluations, ChatGPT-4o1 scored 202 points, ChatGPT-4o scored 180 points, Claude scored 174 points and Gemini scored 110 points (Mert et al., 2025; Thibaut et al., 2024; Traoré et al., 2023). These totals should be interpreted on the full EBHS scale, which ranges from −300 to +300 points. Even the best performing models leave substantial headroom relative to the maximum possible score, and several systems do not reach the pass threshold. This distribution indicates that progress has been made, but further improvements are required before LLMs can reliably cover the full breadth of subspecialty hand surgery knowledge.

Despite these promising results, methodological shortcomings in prior work limit interpretation and practical application. Many earlier studies, including both exam-focused evaluations and broader investigations into the application of AI and LLMs in clinical medicine, education and research, relied on single runs without reporting reliability, used small samples, or were conducted before the release of several 2025 architectures and more competitive open-source systems, limiting their current generalizability. Variability in prompting strategies and reporting practices may have introduced bias, and statistical safeguards such as corrections for multiple comparisons were not consistently applied, complicating between-model inference. Moreover, several investigations addressing AI reliability and performance reproducibility emphasize that systematic analyses of inter-run stability remain uncommon, leaving uncertainty about consistency under repeated testing (Bolgova et al., 2025; Gumilar et al., 2024; Hager et al., 2024; Jung et al., 2023; Lai et al., 2024; Liu et al., 2020; Meskó, 2023; Tam et al., 2024; Wei et al., 2024; Windisch et al., 2024).

To address these gaps, we performed a standardized, multi-model evaluation of 10 contemporary LLMs on the complete 300-statement EBHS written examination, balancing five proprietary and five open-source architectures. In this study, the EBHS Exam is not treated as an examination goal in itself but rather as a controlled-stress test of subspecialty hand-surgery knowledge. By systematically challenging LLMs with standardized, high-stakes content, this format enables identification of clinically relevant failure patterns that may translate into real-world misuse of AI systems in surgical education and informal clinical decision support.

## Material and methods

### Study design

This cross-sectional evaluation assessed the performance of 10 contemporary LLMs on the complete EBHS written examination. Data collection was conducted between October and November 2025. Each model underwent five independent runs across all 300 examination statements, yielding 50 total evaluation sessions and 15,000 individual responses. This multi-run design enabled statistical inference through estimation of inter-run reliability, pairwise performance comparisons and effect size calculation.

A standardized zero-shot evaluation was used to assess pre-trained medical knowledge and to ensure fair comparison across architectures. All procedures were transparently documented to reflect realistic web-interface access, representing how most users interact with these systems. The study followed current best practices for medical AI evaluation and adhered to CONSORT-AI reporting standards (Liu et al., 2020; Rivera et al., 2020; Zaghir et al., 2024).

The study did not involve human participants, patient data or identifiable information; therefore, institutional review board approval was not required.

### LLM selection

Ten LLMs were selected to represent a balanced spectrum of proprietary (privately owned and controlled by specific companies) and open-source architectures, geographic origins and parameter scales.

#### Proprietary models (*n* = 5)

GPT-5 Pro (OpenAI, USA), Claude Sonnet 4.5 (Anthropic, USA), Gemini 2.5 Pro (Google, USA), Grok-4 (xAI, USA), and ERNIE 4.5 Turbo (Baidu, China).

#### Open-source models (*n* = 5)

DeepSeek V3.2 (DeepSeek AI, China), Qwen3 Max (Alibaba Cloud, China), Mistral Medium 3.1 (Mistral AI, France), Llama 3.3 (Meta, USA), and Falcon H1 (Technology Innovation Institute, UAE).

Although the Mistral Medium 3.1 model is a proprietary, hosted system, it originated with a developer whose flagship architectures are open source. To maintain group balance and acknowledge this lineage, the Mistral Medium 3.1 model was classified within the open-source category.

All systems were accessed exclusively through publicly available web interfaces rather than application programming interfaces (APIs), reflecting the predominant interaction patterns of clinical and educational end users. Model versions and timestamps were documented for every evaluation session.

### EBHS examination format

The EBHS written examination comprises 60 multiple-choice questions, each containing five independent true/false statements (300 total binary decisions). The exam covers trauma, nerve surgery, congenital anomalies, microsurgery, degenerative conditions and reconstructive procedures, as described in published reports of the EBHS Examination Committee. A negative-marking scheme applies: +1 for correct, −1 for incorrect and 0 for no answer. Scores range from −300 to +300 points (Calcagni, 2013; Federation of European Societies for Surgery of the Hand; Muir et al., 2018; Siala et al., 2023).

Prior guidance therefore commonly refers to a practical target of 150 points (50% of the maximum) for candidates (Mert et al., 2025). Owing to the +1/−1 scoring, a raw score of 150 points corresponds to 75% accuracy on the 300 items (score = (2 × accuracy − 1) × 300 and accuracy = (score/300 + 1)/2). We therefore report model performance both as a percentage accuracy and as equivalent EBHS points, and we adopt 75% accuracy (150/300 points) as the illustrative pass threshold for this study.

### Prompt engineering methodology

All evaluations used an identical zero-shot prompt across models and runs to assess pre-trained medical knowledge and to minimize elicitation bias. The prompt followed the CO-STAR structure (Context, Objective, Style, Tone, Audience, Response format) to specify task parameters and required concise true/false answers without explanations or hedging. Models were instructed to answer every question and to judge each statement as true or false. The same prompt was used for all sessions. This standardized procedure improved reproducibility and reduced potential confounding from interface behavior or transient model updates, although such sources of variability cannot be fully excluded (Kuerbanjiang et al., 2025; Zaghir et al., 2024).

### Data collection protocol

Data collection took place between October and November 2025 under strict session-independence protocols to ensure the statistical validity of reliability analyses. Each of the 10 models completed five independent runs across all 300 examination statements, yielding 50 sessions and 15,000 binary observations.

Session independence was maintained by starting each run in a new browser session or incognito window, clearing all previous chat history and creating a fresh conversation for every evaluation. Model order was randomized across runs, and timestamps and exact model versions were documented for each session. Data capture involved manual transcription of model outputs from the web-based chat interfaces. Double data entry was performed independently by two researchers.

Models accessed examination content through standardized web chat interfaces rather than APIs, reflecting typical end-user interaction patterns in clinical and educational contexts. Responses were recorded as binary outcomes (1 = correct, 0 = incorrect) based on comparison with the official EBHS answer key. Quality control procedures included verification of response completeness, documentation of any model refusals or formatting inconsistencies, and automated flagging of discrepancies for review.

### Statistical analysis

Statistical analyses were performed in R. A significance threshold of *α* = 0.0011 was applied to control the family-wise error rate after Bonferroni correction for the 45 pairwise model comparisons. Primary outcomes were: (1) mean accuracy per model with 95% confidence intervals computed by the Wilson score method (Wilson, 1927); (2) inter-run reliability quantified by Cohen’s *κ* with interpretation according to Landis and Koch (Landis and Koch, 1977); and (3) pairwise comparisons between models using McNemar’s test for correlated proportions (McNemar, 1947). Cohen’s *κ* was selected for inter-run reliability because the data consisted of binary, item-level decisions, and the primary objective was to assess agreement in responses to identical examination statements rather than stability of aggregated summary scores. Effect sizes were reported as odds ratios with 95% confidence intervals.

For each model, all pairwise *κ* values across the five runs were calculated (10 combinations per model), yielding 100 coefficients in total; the mean *κ* per model summarized overall inter-run reliability. McNemar’s test compared paired responses across the 300 items for each model pair, with exact methods applied when appropriate. All analyses followed predefined protocols and were documented with software and package versions (Gamer et al., 2019; Pocock et al., 2002; Signorell, 2025).

## Results

### Overall model performance

**Figure 1.**
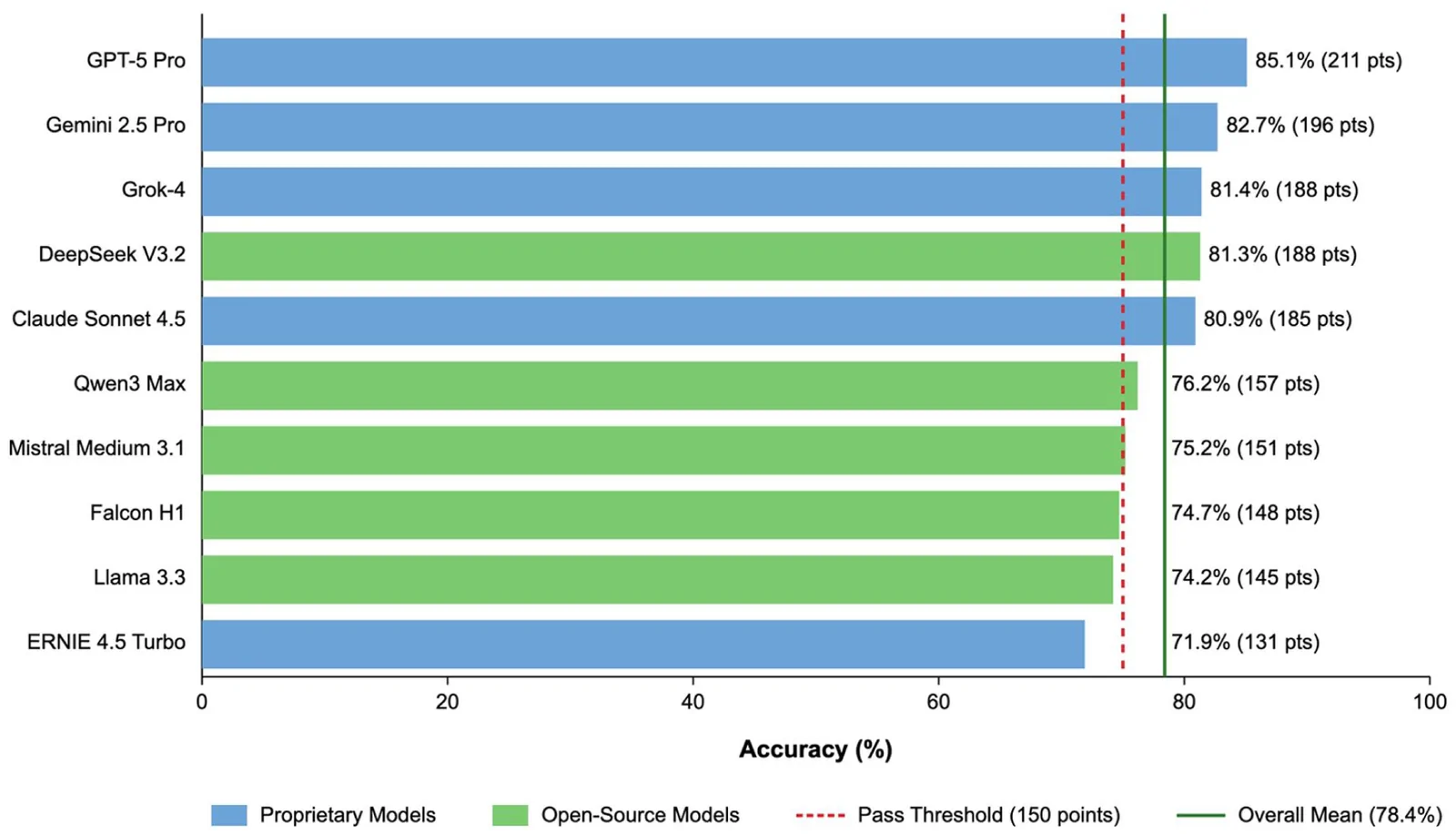
Overall model performance by AI models: mean accuracy (%) and EBHS scoring system (points). Note: mean accuracy (%) for each evaluated model with corresponding EBHS points in parentheses. The red dashed vertical line indicates the illustrative pass threshold used in this study (75% accuracy, equivalent to 150/300 EBHS points). The green vertical line indicates the overall mean accuracy across all models (78 %). Proprietary models are shown in blue and open-source models in green. Abbreviations: AI = artificial intelligence; EBHS = European Board of Hand Surgery; LLM = large language model.

Performance differed among the evaluated systems, with proprietary architectures reaching the higher end results. GPT-5 Pro was the leading model, followed by Gemini 2.5 Pro and Grok-4, while several open-source models approached or, in some cases, reached the adopted passing level. Overall, most systems demonstrated solid baseline competence on the EBHS examination, and the majority reached performance levels compatible with meaningful educational use. Proprietary models generally outperformed open-source architectures, although individual exceptions were observed (Table 1, Figure 1).

**Table 1.** Performance of large language models on the EBHS examination.

Rank	Model	EBHS score (points)	Mean accuracy (%)	95% CI (%)	SD (%)	Cohen's *κ*	Inter-run reliability
1	GPT-5 Pro	211	85	84–86	0.72	0.739	Substantial
2	Gemini 2.5 Pro	196	83	80–86	2.52	0.737	Substantial
3	Grok-4	188	81	80–83	1.02	0.585	Moderate
4	DeepSeek V3.2	188	81	81–82	0.39	0.850	Almost Perfect
5	Claude Sonnet 4.5	185	81	80–82	1.11	0.845	Almost Perfect
6	Qwen3 Max	157	76	76–77	0.47	0.961	Almost Perfect
7	Mistral Medium 3.1	151	75	75–75	0.16	0.979	Almost Perfect
8	Falcon H1	148	75	74–75	0.48	0.965	Almost Perfect
9	Llama 3.3	145	74	74–75	0.39	0.819	Almost Perfect
10	ERNIE 4.5 Turbo	131	72	70–74	1.79	0.731	Substantial

Abbreviations: 95% CI = 95% confidence interval; EBHS = European Board of Hand Surgery.

### Inter-run reliability

**Figure 2.**
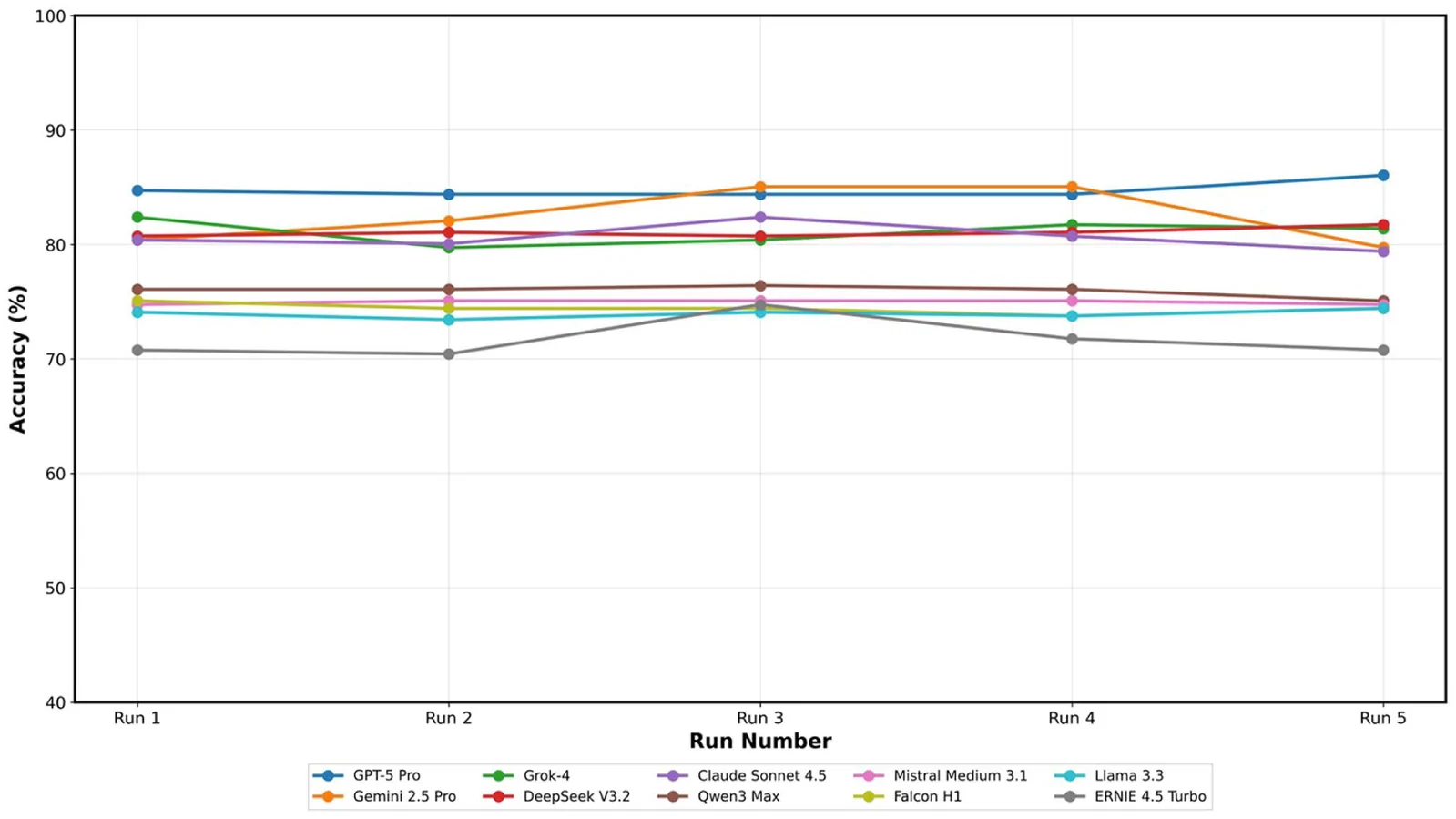
Inter-run consistency across AI models: individual run accuracy for five independent evaluations. Note: Line plot showing model accuracy (%) across five independent evaluation runs. Each coloured line represents one model and markers indicate run-level mean accuracy. The grey dashed horizontal line denotes the illustrative pass threshold (75%). Abbreviations: AI = artificial intelligence.

**Figure 3.**
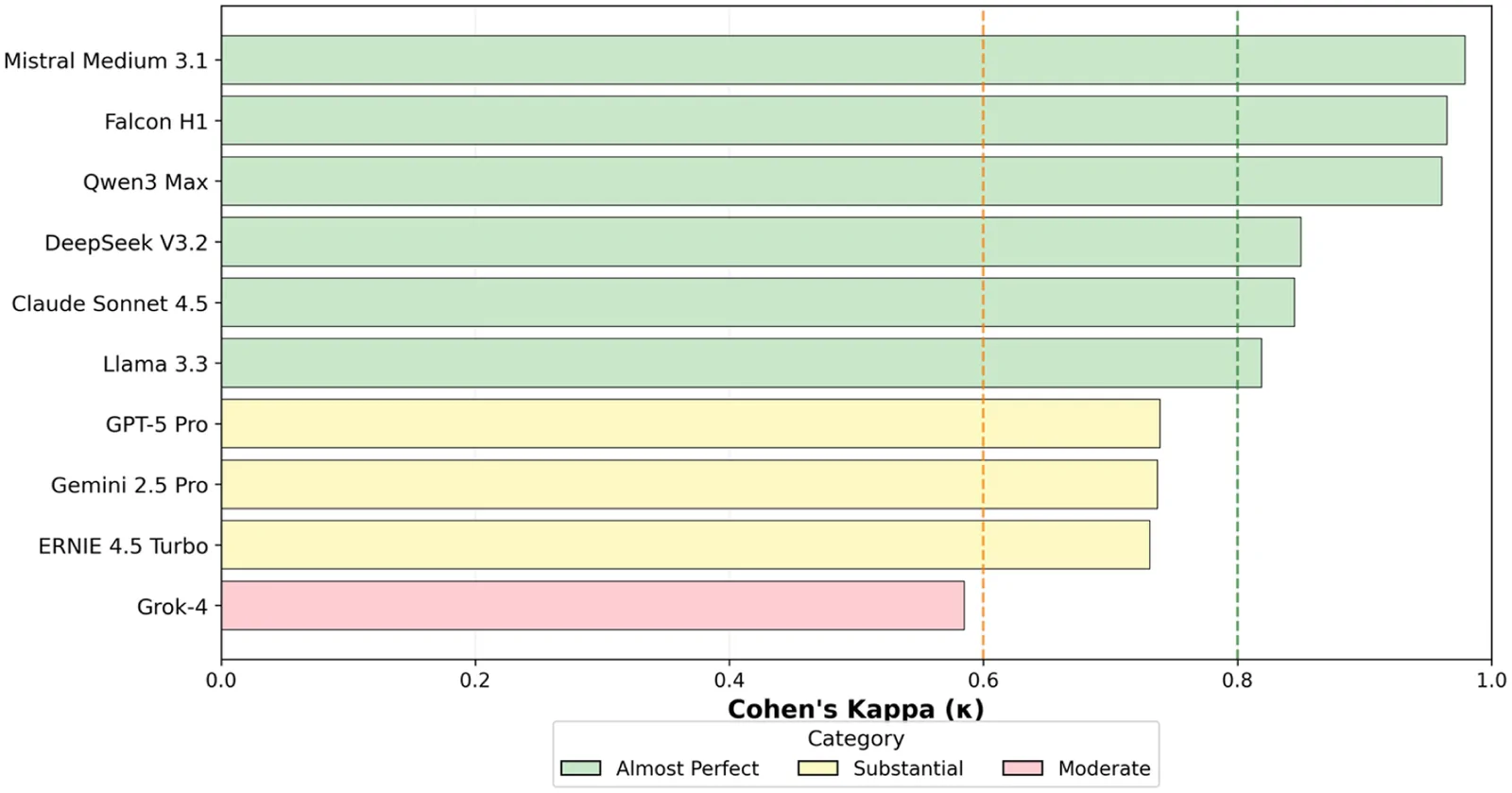
Inter-run reliability (Cohen’s *κ*): agreement between runs for each AI model. Note: Line plot of model-wise Cohen’s kappa coefficients. Dashed vertical lines indicate Landis and Koch interpretation thresholds (*κ* ≥ 0.81 ‘almost perfect’; *κ* ≥ 0.61 ‘substantial’). Corresponding summary values are listed in Table 2 (Landis and Koch, 1977). Abbreviations: AI = Artificial Intelligence; *κ* = Cohen's kappa.

Inter-run reliability was consistently high across the evaluated systems. Most models demonstrated agreement levels consistent with the upper interpretive categories, indicating that repeated evaluations under identical conditions produced stable, reproducible outputs. Only a small minority showed lower but still acceptable consistency. These patterns show that model performance is not driven by stochastic variability alone but reflects stable internal representations of factual knowledge across independent runs (Table 2, Figures 2 and 3).

**Table 2.** Inter-run reliability across models measured by Cohen’s *κ*.

Category (Cohen’s *κ*)	Number of models	Percentage (%)
Almost perfect (0.81–1.00)	6	60
Substantial (0.61–0.80)	3	30
Moderate (0.41–0.60)	1	10
Fair (0.21–0.40)	0	0
Slight (0.00–0.20)	0	0
Poor (<0.00)	0	0
Mean *κ* (SD; range)	0.821 (0.127; 0.585–0.979)	

A clear performance hierarchy was apparent across the full spectrum of evaluated systems, with proprietary models forming the leading tier and open-source architectures generally occupying lower ranks. Pairwise comparisons identified a limited number of statistically significant differences, most of which favoured the highest-performing proprietary systems. Overall, these comparisons reflect a stable ranking structure among models, even though the absolute gaps between systems were moderate and do not limit the potential educational use of several mid-tier architectures (Table 3).

**Table 3.** Pairwise model comparisons using McNemar’s test with Bonferroni correction.

Model A	Model B	Odds ratio	*p*-Value	Interpretation
GPT-5 Pro	Qwen3 Max	3.82	<0.0011	GPT-5 Pro significantly better
GPT-5 Pro	Mistral Medium 3.1	2.89	<0.0011	GPT-5 Pro significantly better
GPT-5 Pro	Falcon H1	3.85	<0.0011	GPT-5 Pro significantly better
GPT-5 Pro	Llama 3.3	4.4	<0.0011	GPT-5 Pro significantly better
GPT-5 Pro	ERNIE 4.5 Turbo	4.08	<0.0011	GPT-5 Pro significantly better
Gemini 2.5 Pro	Falcon H1	2.71	<0.0011	Gemini 2.5 Pro significantly better
Gemini 2.5 Pro	Llama 3.3	2.62	<0.0011	Gemini 2.5 Pro significantly better
Gemini 2.5 Pro	ERNIE 4.5 Turbo	2.53	<0.0011	Gemini 2.5 Pro significantly better

### Question difficulty analysis

Most examination statements were classified as easy based on aggregated model performance, with a smaller proportion requiring intermediate levels of reasoning. Easy items primarily covered fundamental anatomical relationships, straightforward clinical presentations and well-established treatment principles. Medium items typically required nuanced diagnostic distinctions, the selection of appropriate techniques for complex scenarios or the interpretation of ambiguous clinical findings. Hard items were concentrated in specialized domains, including rare congenital anomalies, controversial management strategies, subtle biomechanical principles and questions requiring integration of multiple knowledge domains.

A small subset of questions consistently produced incorrect responses across most or all models, suggesting persistent knowledge gaps shared among architectures. These challenging items frequently involved European-specific practice patterns differing from North American conventions, historical terminology and eponyms, or detailed numerical data from specialized surgical techniques. Overall, the distribution demonstrates broad competency in fundamental hand-surgery knowledge while highlighting ongoing limitations in subspecialty depth and regional practice variability.

## Discussion

This evaluation of 10 contemporary LLMs on the EBHS diploma examination demonstrates robust baseline performance and high inter-run reliability while revealing persistent gaps in subspecialty depth and region-specific knowledge. Overall performance formed a clear ranking across the full model spectrum, with proprietary systems occupying the leading tier and most open-source architectures following at lower ranks. Although several models reached performance levels compatible with structured educational use, a closer look reveals both reproducible errors and inconsistent responses alongside notable weaknesses on highly specialized questions. Taken together, these findings support the potential role of modern LLMs in knowledge consolidation and question validation and formative assessment while underlining the continuing necessity of specialist supervision.

Hard items clustered around rare congenital anomalies, eponyms, complex reconstructions or context-specific surgical details. Notably, these scenarios correspond to clinical situations in which non-specialists or trainees often seek AI-assisted guidance, even though the highest level of subspecialty expertise is required for safe management. A small subset of questions produced incorrect responses across nearly all models, indicating systematic knowledge gaps shared among architectures. These items often reflected European-specific practice patterns, historical terminology or detailed numerical data that are underrepresented in mainstream training corpora. Given that most leading LLMs are trained predominantly on English-language, US-centred data sources, limited exposure to European guidelines and examination standards is likely. For a European subspecialty community, this limitation is particularly relevant and warrants cautious interpretation of LLM-generated outputs in region-specific clinical contexts. The observed distribution therefore reflects broad competence in fundamental hand-surgery knowledge and incomplete coverage of regional, historical and culturally contextual material (Celi et al., 2022; Choi et al., 2025; Schmallenbach et al., 2024). European hand surgeons may therefore need to interpret AI-generated advice with caution when addressing questions governed by European standards or regional practice variations. For candidates preparing for the EBHS examination, this analysis identifies specific topic areas where current LLMs are unreliable.

Across models, responses were frequently reproducible across runs, which may convey a perception of reliability even when subspecialty depth is insufficient. The clinical risk lies less in overtly incorrect answers than in convincingly selected responses that are incomplete or contextually inappropriate. In highly specialized fields such as hand surgery, where decision-making often depends on nuanced anatomical, reconstructive or region-specific considerations, systematic errors may lead to false reassurance rather than error detection, especially in situations where national practice standards differ.

These findings support the view that LLMs should be regarded strictly as decision-support systems rather than autonomous decision authorities. In light of the knowledge gaps identified in this study, the present data underscore that AI-generated outputs require continued specialist oversight and critical appraisal rather than uncritical acceptance.

From an educational perspective, these findings warrant a cautious interpretation of the role of LLMs in hand surgery training. While models with stable behaviour may be used in supervised settings to generate formative question variants or structured self-assessments, their observed performance also exposes important limitations that directly affect learning processes. In the context of postgraduate training, these performance patterns raise concerns regarding knowledge calibration. High accuracy on fundamental concepts combined with systematic deficits in subspecialty depth may lead trainees to overestimate both model reliability and their own level of understanding. This miscalibration represents a subtle but clinically relevant risk, particularly when LLMs are perceived as surrogate supervisors rather than supplementary learning tools. The increasing use of LLMs for clinical documentation, including operative reports, discharge summaries and educational materials, introduces additional medico-legal considerations. Our findings underscore the need for cautious integration of such systems into surgical workflows and for continued expert review of AI-assisted outputs before clinical use or official documentation.

Limitations should be acknowledged. Evaluation through web interfaces may introduce small differences compared with API use but better reflects real-world access. Results represent a single time point and model behaviour may change with subsequent updates. Model versioning remains an important source of variability because provider-side updates can alter accuracy, calibration or response style without explicit disclosure, which limits longitudinal comparability. The Federation of European Societies for Surgery of the Hand did not publish the EBHS items and no model fine-tuning on exam material was performed. However, the possibility that some statements or closely related content appear in public training datasets cannot be entirely ruled out.

Finally, the EBHS examination, while comprehensive in hand surgery, may not generalize to other specialties or to multimodal question formats. Generalization to domains with different curricular structures, imaging-heavy assessments or open-ended tasks is therefore uncertain, and dedicated evaluations across diverse specialties will be required to determine broader applicability.

Future research directions could examine how augmenting LLMs with targeted textbook corpora or curated subspecialty databases affects performance on board-style examinations. Retrieval-augmented generation using authoritative subspecialty resources could address remaining knowledge gaps. Prospective educational trials could measure the real-world impact of AI-assisted learning on trainee performance and confidence calibration. An alternative experimental approach could allow models to abstain from answering when internal uncertainty exceeds a predefined threshold, enabling assessment of confidence calibration. Ideally, future studies could administer a new EBHS examination simultaneously to human candidates and multiple LLMs on the day of the exam, allowing direct comparison under identical conditions before any test content enters public training datasets.

In conclusion, contemporary LLMs demonstrate reliable, reproducible performance on a structured, specialty-focused written examination, but exhibit persistent limitations in depth and region-specific knowledge. Apparent competence on foundational medical knowledge may conceal pronounced deficits in subspecialty knowledge, which risks fostering uncritical reliance during training and everyday clinical practice.

## References

[bibr1-17531934261436305] Bolgova O Shypilova I Mavrych V. Large Language models in biochemistry education: comparative evaluation of performance. JMIR Med Educ. 2025, 11: e67244. 10.2196/67244 PMC12005600 40209205

[bibr2-17531934261436305] Calcagni M . The European board of hand surgery examination. J Hand Surg Eur. 2013, 38: 692–5. 10.1177/1753193413488491 23778070

[bibr3-17531934261436305] Calcagni M. Federation of European Societies for Surgery of the Hand: old and new challenges. J Hand Surg Eur. 2018, 43: 339–40. 10.1177/1753193417748299 29465021

[bibr4-17531934261436305] Celi LA Cellini J Charpignon ML , et al. Sources of bias in artificial intelligence that perpetuate healthcare disparities – a global review. PLOS Digit Health. 2022, 1: e0000022. 10.1371/journal.pdig.0000022 PMC9931338 36812532

[bibr5-17531934261436305] Choi WC Chang CI Choi IC Lam LC. A review of large language models (LLMs) development: a cross-country comparison of the US, China, Europe, UK, India, Japan, South Korea, and Canada. Preprint. 2025. DOI: 10.20944/preprints202504.2136.v1.

[bibr6-17531934261436305] Federation of European Societies for Surgery of the Hand. EBHS Diploma Examination. https://www.fessh.com (24th. November 2025).

[bibr7-17531934261436305] Gamer M Lemon J Fellows I Singh P . irr: various coefficients of interrater reliability and agreement. R package version 0.84.1. 2019. https://CRAN.R-project.org/package=irr (24th November 2025).

[bibr8-17531934261436305] Gilson A Safranek CW Huang T , et al. How does ChatGPT perform on the United States Medical Licensing Examination (USMLE)? The implications of large language models for medical education and knowledge assessment. JMIR Med Educ. 2023, 9: e45312. 10.2196/45312 PMC9947764 36753318

[bibr9-17531934261436305] Gordon M Daniel M Ajiboye A , et al. A scoping review of artificial intelligence in medical education: BEME Guide No. 84. Med Teach. 2024, 46: 446–70. 10.1080/0142159X.2024.2314198 38423127

[bibr10-17531934261436305] Gumilar KE Indraprasta BR Faridzi AS , et al. Assessment of large language models (LLMs) in decision-making support for gynecologic oncology. Comput Struct Biotechnol J. 2024, 23: 4019–26. 10.1016/j.csbj.2024.10.050 PMC11603009 39610903

[bibr11-17531934261436305] Hager P Jungmann F Holland R , et al. Evaluation and mitigation of the limitations of large language models in clinical decision-making. Nat Med. 2024, 30: 2613–22. 10.1038/s41591-024-03097-1 PMC11405275 38965432

[bibr12-17531934261436305] Jung LB Gudera JA Wiegand TLT Allmendinger S Dimitriadis K Koerte IK. ChatGPT passes German State Examination in medicine with picture questions omitted. Dtsch Arztebl Int. 2023, 120: 373–4. 10.3238/arztebl.m2023.0113 PMC10413971 37530052

[bibr13-17531934261436305] Kuerbanjiang W Peng S Jiamaliding Y Yi Y. Performance evaluation of large language models in cervical cancer management based on a standardized questionnaire: comparative study. J Med Internet Res. 2025, 27: e63626. 10.2196/63626 PMC11840365 39908540

[bibr14-17531934261436305] Kung TH Cheatham M Medenilla A , et al. Performance of ChatGPT on USMLE: potential for AI-assisted medical education using large language models. PLOS Digit Health. 2023, 2: e0000198. 10.1371/journal.pdig.0000198 PMC9931230 36812645

[bibr15-17531934261436305] Lai H Ge L Sun M , et al. Assessing the risk of bias in randomized clinical trials with large language models. JAMA Netw Open. 2024, 7: e2412687. 10.1001/jamanetworkopen.2024.12687 PMC11112444 38776081

[bibr16-17531934261436305] Landis JR Koch GG. The measurement of observer agreement for categorical data. Biometrics. 1977, 33: 159–74. 843571

[bibr17-17531934261436305] Lee J Wu AS Li D Kulasegaram KM. Artificial intelligence in undergraduate medical education: a scoping review. Acad Med. 2021, 96: S62–70. 10.1097/ACM.0000000000004291 34348374

[bibr18-17531934261436305] Liu M Okuhara T Chang X , et al. Performance of ChatGPT across different versions in medical licensing examinations worldwide: systematic review and meta-analysis. J Med Internet Res. 2024, 26: e60807. 10.2196/60807 PMC11310649 39052324

[bibr19-17531934261436305] Liu X Cruz Rivera S Moher D Calvert MJ Denniston AK ; SPIRIT-AI | CONSORT-AI Working Group. Reporting guidelines for clinical trial reports for interventions involving artificial intelligence: the CONSORT-AI extension. Nat Med. 2020, 26: 1364–74. 10.1038/s41591-020-1034-x PMC7598943 32908283

[bibr20-17531934261436305] McNemar Q. Note on the sampling error of the difference between correlated proportions or percentages. Psychometrika. 1947, 12: 153–7. 10.1007/BF02295996 20254758

[bibr21-17531934261436305] Mert S Muir L Fuchs B , et al. Can artificial intelligence pass the written European Board of Hand Surgery exam? Hand Surg Rehabil. 2025, 44: 102197. 40447102 10.1016/j.hansur.2025.102197

[bibr22-17531934261436305] Meskó B . Prompt engineering as an important emerging skill for medical professionals: tutorial. J Med Internet Res. 2023, 25: e50638. 10.2196/50638 PMC10585440 37792434

[bibr23-17531934261436305] Muir L Richter M Verstreken F . Eligibility and structures of the European Board of Hand Surgery Diploma exam. J Hand Surg Eur. 2018, 43: 104–6. 10.1177/1753193417740038 29303436

[bibr24-17531934261436305] Nachemson AK. The FESSH Diploma Examination in hand surgery. J Hand Surg Br. 2000, 25: 235–6. 10.1054/jhsb.2000.0428 10961546

[bibr25-17531934261436305] Ouanes K Farhah N. Effectiveness of artificial intelligence (AI) in clinical decision support systems and care delivery. J Med Syst. 2024, 48: 74. 39133332 10.1007/s10916-024-02098-4

[bibr26-17531934261436305] Pocock SJ Assmann SE Enos LE Kasten LE. Subgroup analysis, covariate adjustment and baseline comparisons in clinical trial reporting: current practice and problems. Stat Med. 2002, 21: 2917–30. 10.1002/sim.1296 12325108

[bibr27-17531934261436305] Rivera SC Liu X Chan AW Denniston AK Calvert MJ ; SPIRIT-AI and CONSORT-AI Working Group. Guidelines for clinical trial protocols for interventions involving artificial intelligence: the SPIRIT-AI extension. BMJ. 2020, 370: m3210. 10.1136/bmj.m3210 PMC7490785 32907797

[bibr28-17531934261436305] Schmallenbach L Bärnighausen TW Lerchenmueller MJ. The global geography of artificial intelligence in life science research. Nat Commun. 2024, 15: 7527. 39266506 10.1038/s41467-024-51714-x PMC11392928

[bibr29-17531934261436305] Siala M van der Stok J Durdzinska A Kulkarni K Knie NF. Success in the European Board of Hand Surgery Diploma: candidates’ views from various countries. J Hand Surg Eur. 2023, 48: 598–602. 10.1177/17531934231165409 37066618

[bibr30-17531934261436305] Signorell A . DescTools: tools for descriptive statistics. R package version 0.99.60. 2025. https://CRAN.R-project.org/package=DescTools (accessed 24 November 2025).

[bibr31-17531934261436305] Takagi S Watari T Erabi A Sakaguchi K. Performance of GPT-3.5 and GPT-4 on the Japanese Medical Licensing Examination: comparison study. JMIR Med Educ. 2023, 9: e48002. 10.2196/48002 PMC10365615 37384388

[bibr32-17531934261436305] Tam TYC Sivarajkumar S Kapoor S , et al. A framework for human evaluation of large language models in healthcare derived from literature review. NPJ Digit Med. 2024, 7: 258. 39333376 10.1038/s41746-024-01258-7 PMC11437138

[bibr33-17531934261436305] Thibaut G Dabbagh A Liverneaux P. Does Google’s Bard Chatbot perform better than ChatGPT on the European hand surgery exam? Int Orthop. 2024, 48: 151–8. 10.1007/s00264-023-06034-y 37968408

[bibr34-17531934261436305] Traoré SY Goetsch T Muller B Dabbagh A Liverneaux PA. Is ChatGPT able to pass the first part of the European Board of Hand Surgery diploma examination? Hand Surg Rehabil. 2023, 42: 362–4. 10.1016/j.hansur.2023.06.005 37353199

[bibr35-17531934261436305] Wei Q Yao Z Cui Y Wei B Jin Z Xu X. Evaluation of ChatGPT-generated medical responses: a systematic review and meta-analysis. J Biomed Inform. 2024, 151: 104620. 38462064 10.1016/j.jbi.2024.104620

[bibr36-17531934261436305] Wilson EB. Probable inference, the law of succession, and statistical inference. J Am Stat Assoc. 1927, 22: 209–12.

[bibr37-17531934261436305] Windisch P Dennstädt F Koechli C , et al. The impact of temperature on extracting information from clinical trial publications using large language models. Cureus. 2024, 16: e75748. 10.7759/cureus.75748 PMC11731902 39811231

[bibr38-17531934261436305] Zaghir J Naguib M Bjelogrlic M Névéol A Tannier X Lovis C. Prompt engineering paradigms for medical applications: scoping review. J Med Internet Res. 2024, 26: e60501. 10.2196/60501 PMC11422740 39255030

